# Stress buildup and drop in inland shallow crust caused by the 2011 Tohoku-oki earthquake events

**DOI:** 10.1038/s41598-017-10897-8

**Published:** 2017-08-31

**Authors:** Kiyotoshi Sakaguchi, Tatsuya Yokoyama, Weiren Lin, Noriaki Watanabe

**Affiliations:** 1 Graduate School of Environmental Studies, Tohoku University, Sendai, Japan; 2 OYO Corporation, Saitama, Japan; 3 Graduate School of Engineering, Kyoto University, Kyoto, Japan; 4 Kochi Institute for Core Sample Research, Japan Agency for Marine-Earth Science and Technology, Nankoku, Japan

## Abstract

To examine the change in stress between before and after the Tohoku-oki Mw9.0 earthquake, we performed stress measurements after the earthquake in the Kamaishi mine in Iwate prefecture in northern Japan, located near the northern termination of the mainshock rupture, following previous measurements before the earthquake in the same mine. The results showed that the magnitudes of the three-dimensional principal stresses and the vertical stress drastically increased after the mainshock and, at 1 year after the earthquake, were more than double those before the earthquake. The principal stress magnitudes then decreased with time and returned to almost pre-earthquake levels at about 3 years after the earthquake. These changes can be interpreted in terms of coseismic rupture of the mainshock and the occurrence of aftershocks in the Sanriku-oki low-seismicity region (SLSR), where the Kamaishi mine is located. The drastic increase in stress suggests that the SLSR may act as a barrier to further rupture propagation.

## Introduction

In general, stress and earthquakes are known to be interrelated: stress triggers earthquakes and earthquakes alter the shear and normal stresses in surrounding faults^1–5^. During the 2011 Tohoku-oki, Japan, earthquake (Mw 9.0), rupture of the plate boundary fault between the Pacific plate and the North American plate caused a distinct decrease in stress in the earthquake source region^6–9^. Stress states outside the earthquake source region may differ from those inside the area. Understanding of the stress state outside an earthquake source region both before and after a great earthquake may provide important evidence to explain how rupture propagation behaved outside the source region during the event.

**Figure 1:**
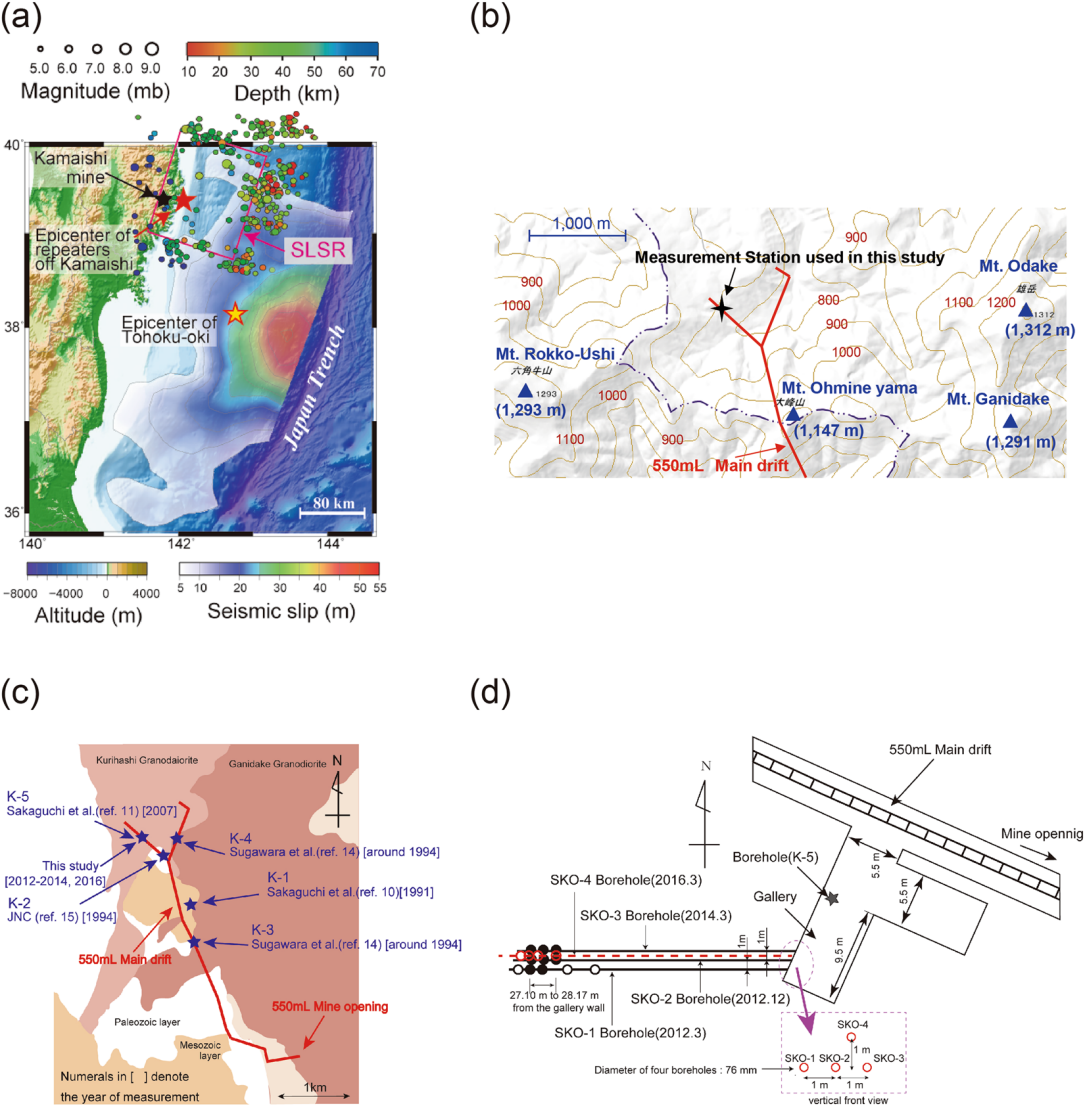
Maps of the measurement location. (**a**) The positional relationship between the Kamaishi mine and the epicentre of the Tohoku-oki earthquake (yellow star with a red outline). The epicentre of repeaters off Kamaishi is marked by a red star and the location of the Kamaishi mine is indicated by a black star (modified from ref. 34). The total slip distribution of slip greater than 5 m for the 2011 Tohoku-oki earthquake is also plotted, from Yagi and Fukuhata^16^ and the epicentres of earthquakes (M > 5) in the Sanriku-oki low-seismicity region (SLSR), from Ye *et al*.^33^. (**b**) Topographical map of the area around the Kamaishi mine, created by the first author based on a topographic map. (Geospatial Information Authority of Japan; http://mapps.gsi.go.jp/maplibSearch.do#1) (**c**) Plan view of the drift at the 550 mL site at the Kamaishi mine. The measurement station used in this study and those in other studies with the Compact Conical-ended Borehole Overcoring (CCBO) technique are indicated by stars. In this Fig., K-1 denotes the measurement station used by Sakaguchi *et al*.^10^, K-2 the station used by JNC (Japan Nuclear Cycle Development Institute, current Japan Atomic Energy Agency: JAEA)^15^ and K-3 and K-4 denote those used by Sugawara *et al*.^14^. K-5 marks the measurement station used by Sakaguchi *et al*.^11^ with the Downward Compact Conical-ended Borehole Overcoring (DCCBO) technique^12, 13^, which is located in the vicinity of the measurement station in this study. The DCCBO technique is an improved version of the CCBO technique that can be applied to a vertical borehole from the surface. (This figure was generated using Adobe Illustrator software, version number Illustrator CS5 15.1.0) (**d**) Plan view of the measurement station. The measurement station is located where two galleries (width ~5.5 m, height ~7 m) are adjacent. *in situ* stress measurements were performed in four boreholes (SKO-1, SKO-2, SKO-3 and SKO-4), which are denoted by thick solid lines and a red broken line. The star in this figure indicates the K-5 measurement borehole used by Sakaguchi *et al*.^11^ with the DCCBO technique using a downward borehole from the gallery floor.

The determination of the absolute magnitude of stress is also an important consideration in engineering fields that involve the Earth’s crust, such as exploration for oil and natural gas, the extraction of geothermal energy and deep mining at depths of several kilometres. Moreover, *in situ* rock stress is an important concern in the construction of tunnels and underground caverns at depths of tens or even several hundreds of metres. For such purposes, several *in situ* rock stress measurements have been performed at the Kamaishi mine in northeast Japan (Fig. 1(a)) from 1991 to 2007^10–15^. The coseismic slip of the plate interface below the Kamaishi region during the Tohoku-oki earthquake was relatively slight^16–19^ (Fig. 1(a)), and this may have acted as a barrier to further rupture propagation during the earthquake. Generally, Coulomb stress change is used to estimate the stress field after an earthquake; for example, Toda *et al*.^20^, Hiratsuka and Sato^21^ and Sato *et al*.^22^ reported preliminary results for the Coulomb stress change after the Tohoku-oki earthquake. However, the Coulomb stress is an estimate of the stress difference based on the slip distribution assuming the target fault. Therefore, it is important to clarify the change in the absolute magnitude of stress before and after the earthquake. For this purpose, the results of stress measurements before the earthquake are indispensable, because they cannot be obtained after the earthquake. The stress measurements prior to the Tohoku-oki earthquake provided us with a unique opportunity to examine the change in stress in the Kamaishi mine before and after the Tohoku-oki earthquake. Consequently, we regularly repeated stress measurements at the same research site in the Kamaishi mine after the earthquake using the same method as studies conducted prior to the earthquake.

## Results

### Research site and stress measurements

The Kamaishi mine was originally developed as an underground metal mine and is not currently operational. Its main drifts and galleries are now maintained as a source of mineral water for drinking and are also used for scientific research. This mine is located about 170 km northwest of the Tohoku-oki earthquake epicenter. Coseismic slip of the plate fault beneath the onshore Kamaishi region and the offshore Kamaishi region (hereafter the Kamaishi regions) during the Tohoku earthquake can be considered to be quite small based on the shape of the seismic slip contour line of 5 m (Fig. 1(a)). It has been reported that the coseismic surface crustal movement during the earthquake in this region consisted of horizontal movement (to the east-southeast) of 3.32 m and subsidence of 0.5 m^23^. In this study, we selected a measurement station(39°19′N, 141°40′E) that is about 5 km from the mine opening at the 550 mL site at the Kamaishi mine (Fig. 1(b and c)). The stress state around this measurement station is not affected by galleries or goaf caverns because the measurement points are sufficiently far from the galleries and/or caverns. This study area is near the centre of the Kurihashi granodiorite body. The Kurihashi granodiorite is one of several Early Cretaceous (120–110 Ma) granite bodies intruded into the Palaeozoic–Mesozoic sedimentary rocks in the Kitakami area^24^. The mean values of the Young’s modulus, Poisson’s ratio and the unit volume weight of this rock at the measurement station are 52 GPa, 0.17 and 27 kN/m^3^, respectively. Additionally, there are no active faults in the region shown in Fig. 1(b) [Active fault database of Japan, https://gbank.gsj.jp/activefault/index_e_gmap.html]. Furthermore, no seismicity was detected within 15 km of the mine in the seismicity observations conducted by JNC between 1990 and 1998^15^.

The stress measurement stations (K-1 to K-5) used both before and after the Tohoku-oki earthquake occur within a distance of about 1.5 km (north to south; Fig. 1(c)). The overburden at this measurement station is about 290 m. We performed stress measurements four times after the earthquake. The first measurement (SKO-1) was carried out from February 27 to March 1, 2012, the second (SKO-2) from December 17 to 19, 2012, the third (SKO-3) from March 10 to 12, 2014 and the fourth (SKO-4) from March 14 to 17, 2016, approximately 1, 2, 3 and 5 years after the earthquake, respectively. The four horizontal boreholes used for stress measurements (SKO-1, 2, 3 and 4) were aligned in parallel at 1-m intervals (Fig. 1(d)). For each borehole, we performed three to five measurements.

Measurements were performed using the Compact Conical-ended Borehole Overcoring (CCBO) technique^14, 25^. The CCBO technique is a type of overcoring, which is a stress relief method. In this method, a suite of strain sensors (or displacement sensors) is first set on the wall or the bottom of a borehole, and then overcoring, which is core-boring to isolate a rock sample and strain sensors from the rock mass to relieve *in situ* stress, is conducted. The relieved strain/displacement that accompanies the overcoring process is monitored continuously. The absolute value of a three-dimensional stress tensor can then be determined based on the elastic theory using the final relieved strain. The CCBO technique is an improved method that offers greater accuracy and economy; it has been suggested by the International Society for Rock Mechanics (ISRM)^14^ and is also authorised as a standard method of the Japanese Geotechnical Society (JGS)^26^. The CCBO technique has been applied in Japan and in other countries^27–30^. Details of the measurement methods used in this study are provided in the Methods section (CCBO technique) and Supplementary Information (Supplementary Figure 1).

**Figure 2:**
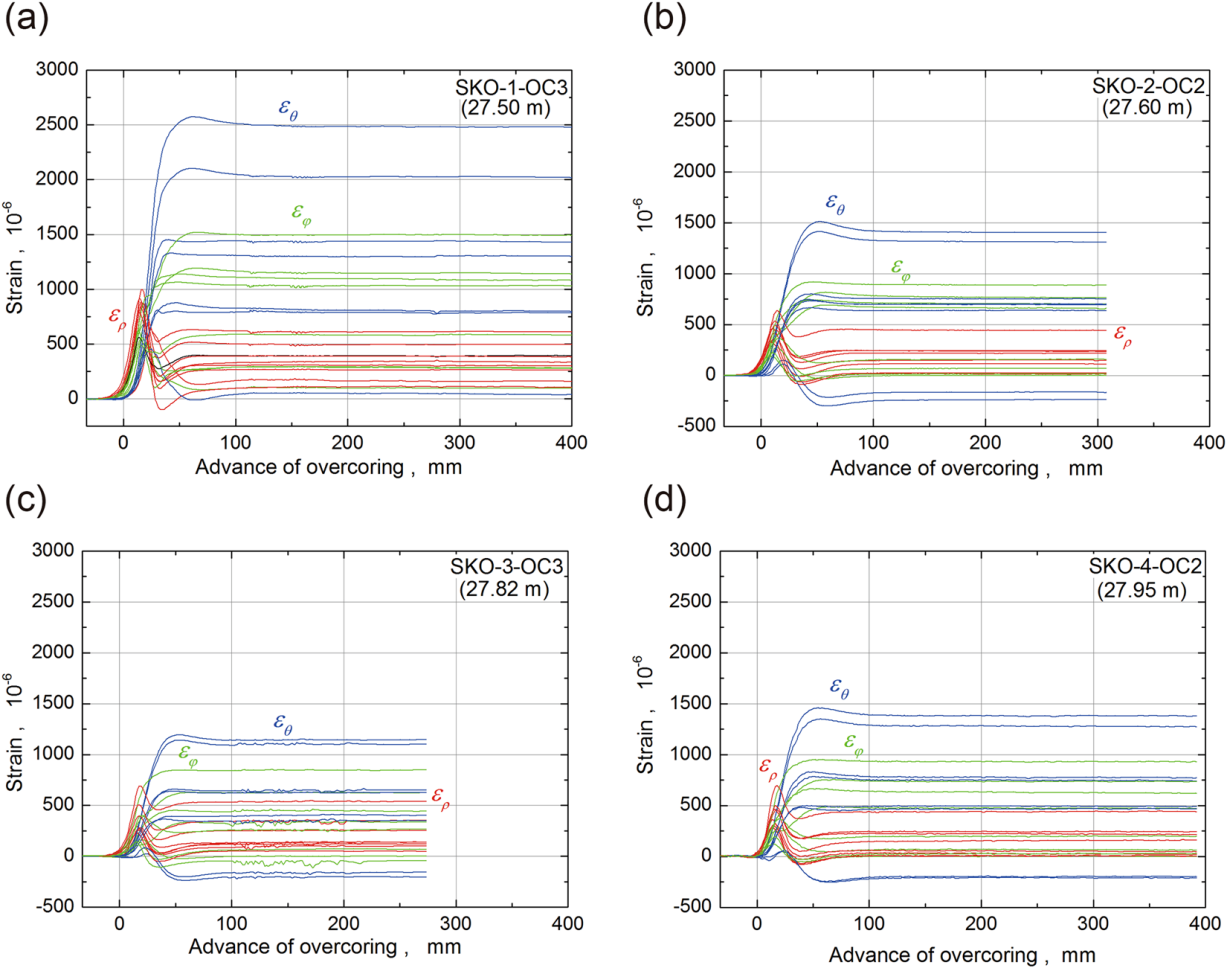
Examples of the strain response during overcoring. The x-axis is the advance of overcoring, and zero on the x-axis corresponds to the position of strain gauges on the strain sensor for the CCBO technique. Strains of the *θ*-axis are denoted by blue lines, *ρ*-axis strains by red lines and *φ*-axis strains by green lines. (**a**) Results for SKO-1-OC3. (**b**) Results for SKO-2-OC2. (**c**) Results for SKO-3-OC3. (**d**) Results for SKO-4-OC2.

We monitored the elastic strain in a total of 24 directions using eight rosette-type strain gauges (with three components in three directions) during the overcoring process (Fig. 2). Although the depth from the gallery wall and the position of each strain gauge in the borehole socket were almost the same, the strain values for SKO-1 (~1 year after the mainshock) were greater than those of the other three boreholes, and the strains for SKO-2 (~2 years after the mainshock) were greater than those for SKO-3 (~3 years after the mainshock). Moreover, the strain for SKO-4 (~5 years after the mainshock) was the same as that for SKO-2. This result suggests that the stress level at the time of measurement decreased in the order SKO-1, 2, 4 and 3, because the lithology was similar for all boreholes. The strain curves shown in Fig. 2 are for only one measurement point for each of SKO-1, 2, 3 and 4; however, a similar trend was observed for the other measurement points. Moreover, the shapes of strain (*ε*_*ρ*_, *ε*_*θ*_, *ε*_*φ*_) in three typical directions (see Supplementary Figure 2 for the details) during the overcoring process are consistent with the theoretical shape based on the elastic theory^14^ (see Supplementary Figure 3 for the details).

### Orientation of the principal stresses

**Figure 3:**
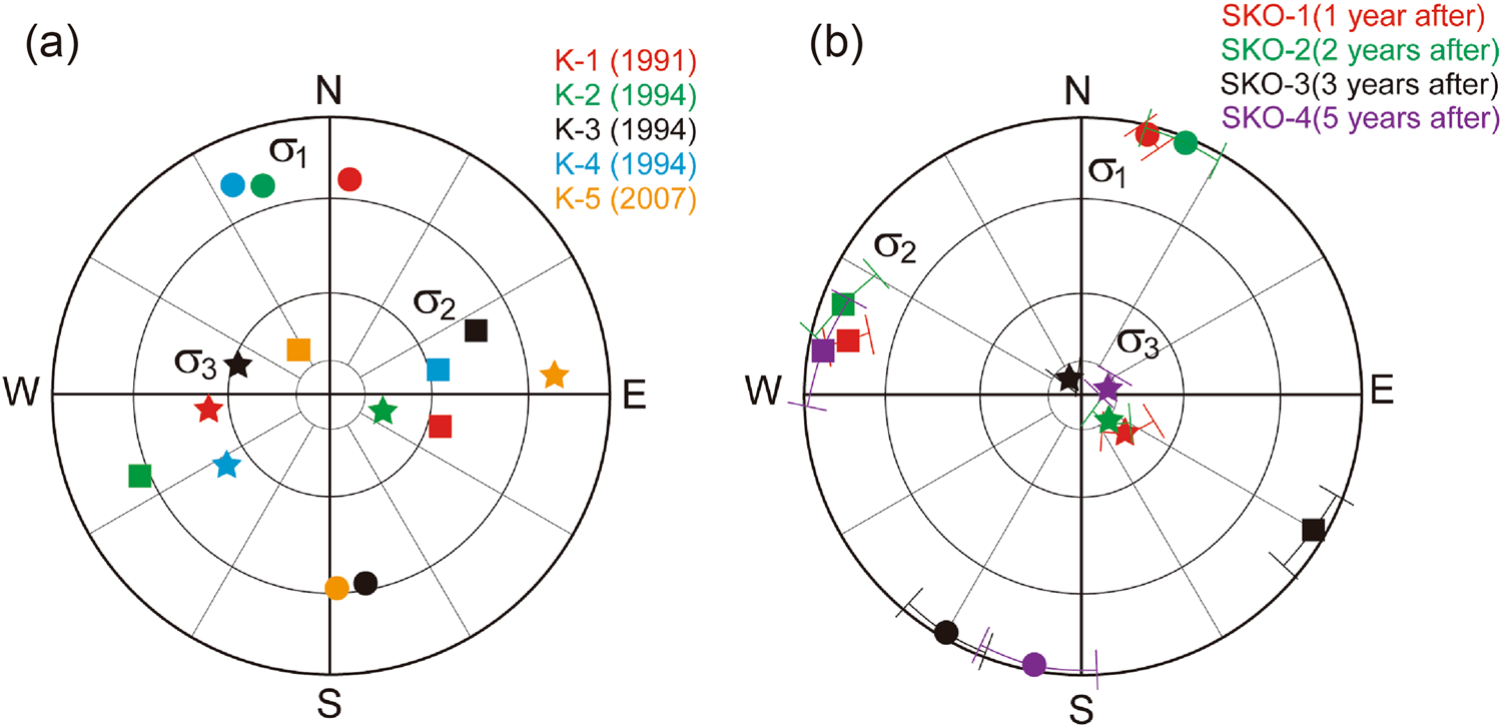
Orientation of the principal stresses (*σ*_1_, *σ*_2_, *σ*_3_) on a lower-hemisphere stereographic projection. In the projection, *σ*_1_ (circles) denotes the maximum principal stress, *σ*_2_ (squares) the intermediate principal stress and *σ*_3_ (stars) the minimum principal stress. (**a**) Results obtained before the earthquake (K-1 to K-5). (**b**) Results from 1 year after to 5 years after the earthquake. These results are the mean values. These mean values of the direction of principal stresses were calculated by averaging the values of the principal stresses at every measuring point.

The maximum principal stress *σ*_1_ determined in this study was oriented north-south both before and after the Tohoku-oki earthquake (Fig. 3). This finding is consistent with previous studies regarding the *σ*_1_ orientation in the Kamaishi mine^31^. In contrast, both the intermediate principal stress *σ*_2_ and the minimum principal stress *σ*_3_ show clear differences before and after the earthquake. The intermediate principal stress *σ*_2_ of the measurements, with the exception of K-2, had a steep dip angle more than 30° before the earthquake but was almost horizontal after the earthquake. Throughout the four measurements after the earthquake, the intermediate principal stress *σ*_2_ was in an east-west horizontal direction with very small dip angles, and the minimum principal stress *σ*_3_ was vertical. Overall, the stress states both before and after the earthquake are consistent with a reverse-faulting stress regime, which is consistent with the foreshocks and aftershocks in the Kamaishi regions^32^. This regime was maintained for up to the last measurement at ~5 years after the earthquake.

### Magnitudes of the principal stresses and the overburden pressure

**Figure 4:**
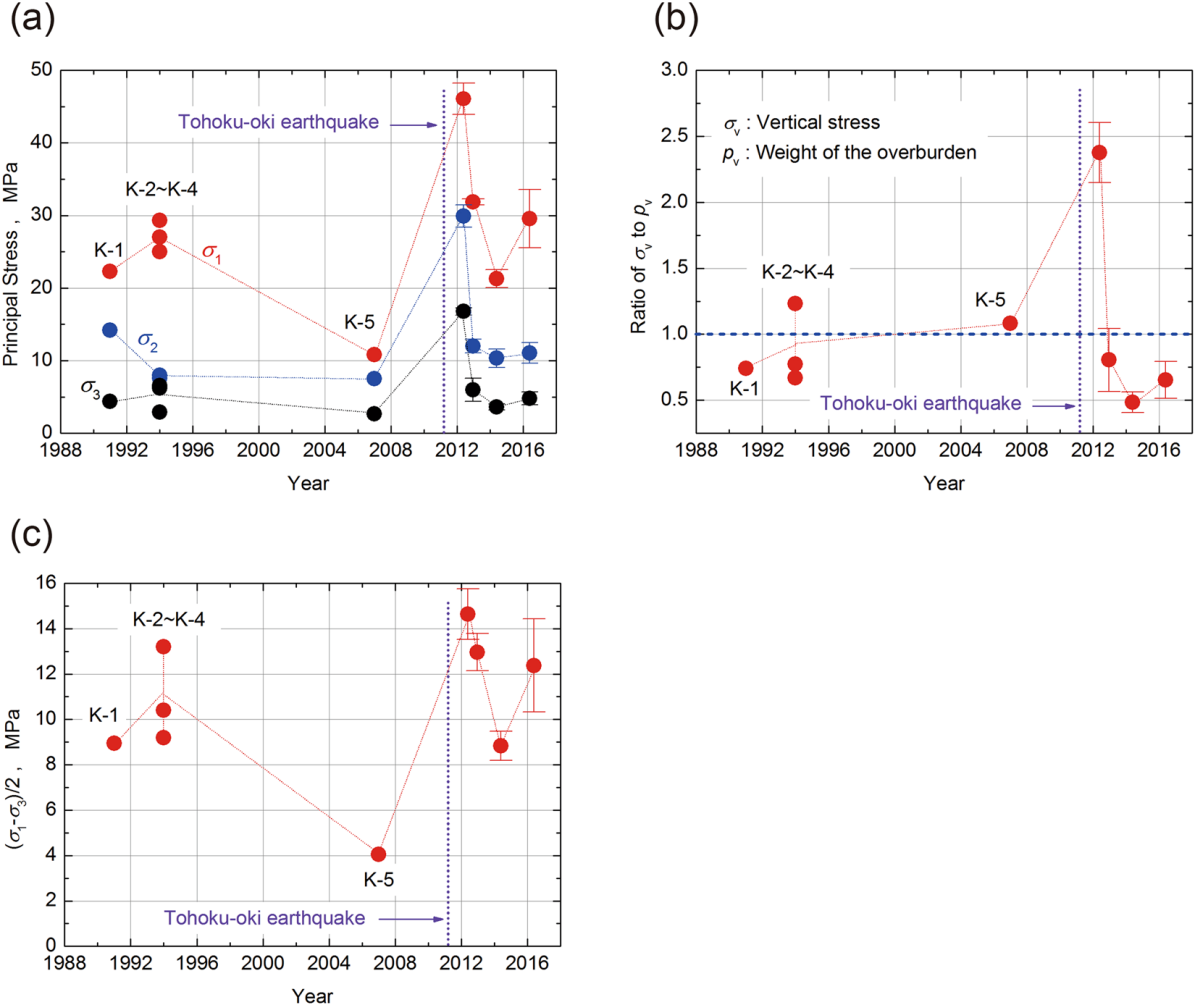
Annual trends in the stress state. The results after the Tohoku-oki earthquake are mean values. (**a**) The three principal stress magnitudes (*σ*_1_, *σ*_2_, *σ*_3_). These mean values of principal stresses were calculated by averaging the principal stress at every measuring point. (**b**) The ratio of the vertical stress *p*_v_ to the overburden pressure *p*_v_ calculated as (gravitational acceleration) × (depth) × (average density determined from rock samples to be 2.7 ton/m^3^). The error of overburden pressure *p*_v_ was evaluated assuming that the measured value of overburden (depth) has an error ±10 m. (**c**) The maximum shear stress (=(*σ*_1_ − *σ*_3_)/2).

There is a clear difference in the magnitudes of the principal stresses before and after the earthquake (Fig. 4(a)). The magnitudes of the principal stresses 1 year post-earthquake were two to three times greater than those before the earthquake. However, 2 years after the earthquake, the magnitudes of the intermediate principal stress and the minimum principal stress were almost the same as those before the earthquake, whereas the magnitude of the maximum principal stress was similar to the values in 1991 and 1994 but still larger than that in 2007, which was thought to entail some uncertainty. The magnitude of the maximum principal stresses 3 years after the earthquake was smaller than the values in 1991 and 1994. However, 5 years after the earthquake, the magnitudes of principal stresses had returned to the same level as in 1991 and 1994.

Figure 4(b) shows the ratio of the vertical stress *σ*_v_ to the overburden pressure *p*_v_ as estimated from the rock density and the depth from the ground surface just above the measurement station. The magnitude of the vertical stress *σ*_v_ was almost the same as the overburden pressure except at 1 year after the earthquake: the magnitude of *σ*_v_ at 1 year post-earthquake was approximately 2.4 times greater than the overburden pressure (Fig. 4(b)). Theoretically, the static vertical stress has to be in a state of mechanical equilibrium with the overburden pressure. In the study area, the topography is steep: three tall mountains with altitudes of approximate 1300 m are located within 3 km of the stress measurement station (Fig. 1(b)). These three mountains are approximately 300–400 m higher than the ground surface immediately above the measurement station. As the height difference is larger than the overburden ~290 m above the measurement station, a higher vertical stress magnitude of more than two times of the overburden pressure can be considered to be possible for a short period of time (1 or 2 years).

### Maximum shear stress

From the measured maximum and minimum stress magnitudes, we calculated the maximum shear stress, *τ*_max_ = (*σ*_1_ − *σ*_3_)/2 (Fig. 4(c)). As with the three principal stresses, the magnitude of the maximum shear stress increased as a result of the Tohoku-oki mainshock, decreased until 3 years after the earthquake and subsequently returned to an increasing trend 5 years after the earthquake. This shear stress measured in 2007, which showed a small value, was probably affected by the uncertainty of the maximum principal stress value in the 2007 measurement, for which a vertical borehole was used, in contrast to the horizontal boreholes for the measurements in other years.

In summary, the results (K-2, 3, 4) in the same year (1994) exhibited good repeatability with regard to orientation and magnitude; in addition, the results of repeated measurements from 1991 to 2016 showed reasonable trends, good repeatability and sufficient precision (Figs 3 and 4). Therefore, we conclude that these results are reliable and correct, despite exhibiting some scatter.

## Discussion

The Kamaishi regions (both onshore and offshore) were called the Sanriku-oki low-seismicity region (SLSR), which is located near the northern termination of the Tohoku-oki mainshock rupture, by Ye *et al*.^33^ (pink rectangular frame in Fig. 1(a)). The SLSR lacks historical great earthquake e.g. greater than M7 class earthquakes and has shown relatively low levels of moderate-size earthquakes (*M* = 4.7–5.1) over the ~50 years before the 2011 Tohoku-oki earthquake at an average interval of 5.5 years^33, 34^. However, earthquakes in the SLSR suddenly became much more frequent after the Tohoku-oki mainshock, and the magnitudes of those earthquakes increased^34^. Furthermore, the interval between earthquakes off Kamaishi gradually increased with time after the Tohoku-oki mainshock, from about ten days in the first month after the mainshock (three M5.5–5.9 aftershock occurred in one month) to approximately 0.3 years in the first year after the mainshock. At the same time, the magnitudes of these earthquakes returned to the same levels as those before the mainshock.

Our stress measurements after the Tohoku-oki earthquake and those obtained in previous studies in 1991–2007 in the Kamaishi mine showed that the magnitudes of the three principal stresses and the maximum shear stress rose suddenly just after the mainshock, then gradually decreased and returned to almost the same levels as those before the mainshock within approximately 5 years.

Although the depth of our stress measurements was much shallower than the plate boundary fault, which is at about 50 km depth, from the results of the stress measurements and the seismicity in the Kamaishi regions before and after the Tohoku-oki mainshock, we can suggest that the stress magnitudes in these regions suddenly increased because the Kamaishi regions delimit the northern extent of the Tohoku-oki mainshock rupture. In other words, the Kamaishi regions probably acted as a barrier to further rupture propagation during the Tohoku-oki mainshock. As a result of the sudden and dramatic increase in stress, earthquakes in offshore Kamaishi became much more frequent and stronger just after the mainshock. These earthquakes led to a decrease in the magnitude of stress in the Kamaishi regions after 1 year following the mainshock. Finally, the stress magnitudes returned to almost the same levels as those before the Tohoku-oki mainshock within 2–3 years, accompanied by a decrease in the frequency of aftershocks in offshore Kamaishi. Iinuma *et al*.^35^ showed that the cumulative postseismic slip (for the period from 23 April 2011 to 10 December 2011) of the 2011 Tohoku-oki earthquake in offshore Kamaishi was larger than that of surrounding regions. Moreover, Toda *et al*.^20^, Hiratsuka and Sato^21^ and Sato *et al*.^22^ showed that the Coulomb stress change (ΔCFF) around the Kamaishi mine exhibited a positive trend after the Tohoku-oki earthquake, and Bletery *et al*.^36^ demonstrated that the stress drop altered to a negative trend after the mainshock (see Supplementary Figure 8). Additionally, Ishibe *et al*.^37^ showed that the temporal changes in median ΔCFF from 2000 to the middle of 2015. The median values of ΔCFF rapidly increased just after the Tohoku-oki mainshock, after which the median ΔCFF gradually decreased to background levels approximately 3 years after the mainshock. Uchida *et al*.^38^ showed that the stress drop due to earthquakes off Kamaishi (20 Mar. 2011–23 Sep. 2011) was 2.4 MPa to 10.4 MPa. These observations also support our interpretation of the stress change pattern examined in this study. In other words, the build up and down of stress measured in the Kamaishi mine has been influenced by the both the mainshock and the aftershock occurred off Kamaishi. That is: i) the stress magnitude in the Kamaishi region drastically increased during the Tohoku-oki mainshock because the region probably acted as a barrier to further rupture propagation; ii) the increased stress caused earthquakes in offshore Kamaishi to occur more actively in terms of both frequency and magnitude (Ye *et al*.^33^ and Ariyoshi *et al*.^34^); and therefore, iii) the frequent aftershock occurrence cased the stress in Kamaishi mine to decrease and to return to the approximately same level as before the 2011 Tohoku-oki earthquake (Ishibe *et al*.^37^) as well as increasing the cumulative postseismic slip in this region (Iinuma *et al*.^35^).

In addition, the consistency between the change in measured stress and the change in seismicity in the Kamaishi regions suggests that the results of stress measurements, even those at a much shallower depth than the earthquake source fault, can be useful for understanding rupture-propagation behavior.

## Methods

### The CCBO technique

The procedures used to measure *in situ* stress with the CCBO technique are illustrated in Supplementary Figure 1.

We conducted five, three, three and four measurements at 1, 2, 3 and 5 years after the Tohoku-oki earthquake, respectively (see Fig. 1(d) in the main text). The measurements in the SKO-1 borehole were performed at five measurement points located 20.06 m to 28.56 m from the gallery wall. However, because of the possible influence of the gallery, the results at 20.06 m (SKO-1-OC1) and 23.57 m (SKO-1-OC2) were excluded from the analysis. The results at 28.56 m (SKO-1-OC5) were also excluded, because of the lack of a borehole socket based on the appearance of the recovered core after measurement. The measurements in the SKO-2 borehole were performed at three measurement points: 27.10 m (SKO-2-OC1), 27.60 m (SKO-2-OC2) and 28.03 m (SKO-2-OC3) from the gallery wall. The measurements in the SKO-3 borehole were performed at three measurement points: 27.10 m (SKO-3-OC1), 27.82 m (SKO-3-OC2) and 28.17 m (SKO-3-OC3). The measurements in the SKO-4 borehole were performed at four measurement points: 27.11 m (SKO-4-OC1), 27.95 m (SKO-4-OC2), 28.52 m (SKO-4-OC3) and 28.98 m (SKO-4-OC4). However, because of the technical problem, the result at 27.11 m (SKO-4-OC1) was excluded from the analysis. There were no problems with any of the other measurements.

### Estimation of Young’s modulus and Poisson’s ratio

The Young’s modulus and Poisson’s ratio were determined by a multi-stage uniaxial compression test^26^. The specimens were prepared from the recovered core with a strain cell (Supplementary Figure 4). Three mutually perpendicular cylindrical rock-core specimens 25 mm in diameter and 50 mm in length were prepared by drilling the recovered core with the strain cell. Two cross-type strain gauges were used to measure the strain response of each specimen. The strain gauges were bonded to the cylindrical surface of the specimens at a height of 12.5 mm at an offset of 180°. Cyclic loading and unloading was performed five to seven times at a loading rate of about 0.1–0.2 MPa/sec (Supplementary Figure 5). The maximum load during the loading cycle was set to twice the overburden pressure at the measurement station. The Young’s modulus and Poisson’s ratio were determined from the stress–strain relation (Supplementary Figure 6) and the linear relation between the axial stress and the strain recovery (Supplementary Figure 7). Supplementary Figure 7 was drawn as follows: the axial stress and recovered axial and lateral strains from the beginning to the end of unloading were read in each cyclic loading and plotted as shown in Supplementary Figure 6. The data were subjected to the least-squares method to determine the gradients of stress: axial and lateral strain. The slope of the line approximated from the axial stresses and strains is defined as the Young’s modulus, and the ratio of the slope of the axial relation to that of the lateral relation is defined as the Poisson’s ratio.

### Anisotropy of Young’s modulus and Poisson’s ratio

The degree of anisotropy of the Young’s modulus was 1.5%–8.2%; in contrast, the Poisson’s ratio did not show anisotropy. We used the mean values of the Young’s modulus and Poisson’s ratio at each measurement point to evaluate the *in situ* stress under the assumption of isotropy. Therefore, the evaluated stress in this study includes approximately a few percent uncertainty.

### Data availability

Data supporting Figs 3 and 4 are available in Supplementary Information Tables 2–6.

## Electronic supplementary material

Supplementary information 
