# Supplementary material for: Stress buildup and drop in inland shallow crust caused by the 2011 Tohoku-oki earthquake events
Source: Sci Rep. 2017 Aug 31;7:10242. doi: 10.1038/s41598-017-10897-8 (PMC5579283; doi:10.1038/s41598-017-10897-8)
Supplement: Supplementary file 1 — Supplementary information [file 41598_2017_10897_MOESM1_ESM.pdf]

Supplementary Information

Title

Stress buildup and drop in inland shallow crust caused by the 2011 Tohoku-oki earthquake events

Author names and affiliations

Kiyotoshi Sakaguchi[1*], Tatsuya Yokoyama[2], Weiren Lin[3, 4] and Noriaki Watanabe[1]

[1] Graduate School of Environmental Studies, Tohoku University, Sendai, Japan

[2] OYO Corporation, Saitama, Japan

[3] Graduate School of Engineering, Kyoto University, Kyoto, Japan

[4] Kochi Institute for Core Sample Research, Japan Agency for Marine-Earth Science and Technology, Nankoku, Japan

[*]Correspondence and requests for materials should be addressed to K. S. kiyotoshi.sakaguchi.c5@tohoku.ac.jp

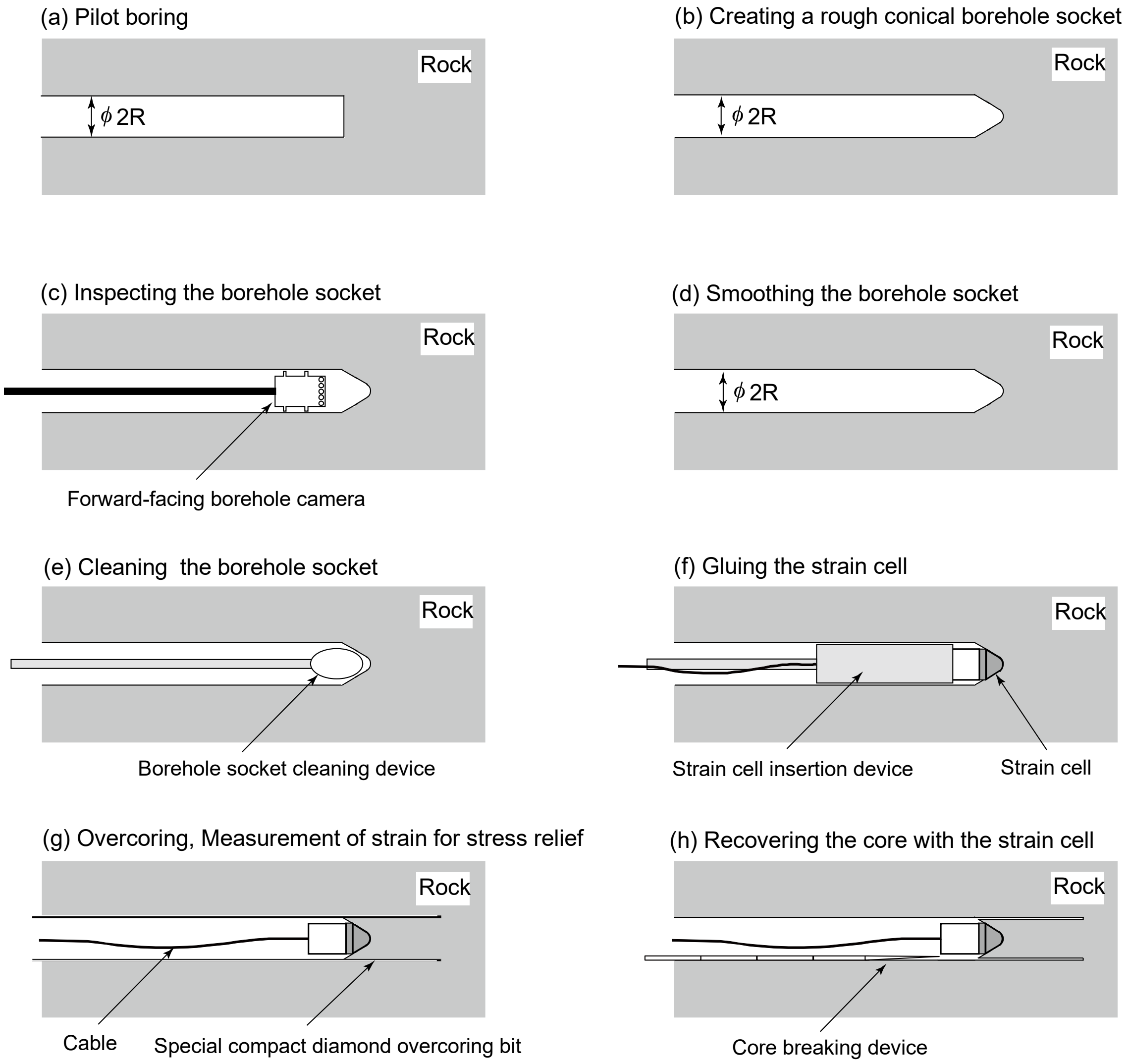

Supplementary Figure 1 Steps for in-situ stress measurement using the Compact Conical-ended Borehole Overcoring (CCBO) technique applied in this study [1]. (a) A horizontal pilot borehole ($2R$ = 76 mm) with coring was drilled to the measurement point from the gallery wall. (b) A roughly conical borehole socket was created using a special-purpose diamond bit

with a borz crown. (c) The borehole socket was inspected using a forward-facing borehole camera. (d) The borehole socket was smoothed using an impregnated special-purpose diamond bit. (e) The borehole socket was cleaned using a soft cloth and acetone attached to a cleaning device. (f) A 24-element strain cell, on the surface of which eight rosette-type strain gauges were axisymmetrically and equally spaced along a circle of radius $0.5R$ = 19 mm was attached to the insertion device. Adhesive was then spread over the head of the strain cell. The strain cell was inserted into the borehole and pushed forward to the socket until the adhesive had hardened. The rotation angle of the installed strain cell was recorded at the same time. (g) Overcoring was conducted using a special compact diamond overcoring bit with an outer diameter of 76 mm and an inner diameter of 70 mm. The overcoring length was 300–400 mm. During overcoring, progress was monitored using a displacement transducer, and strains and displacement were recorded at every 3 mm of overcoring advance using data-logging equipment and a PC. (h) After overcoring, the core with the strain cell was recovered.

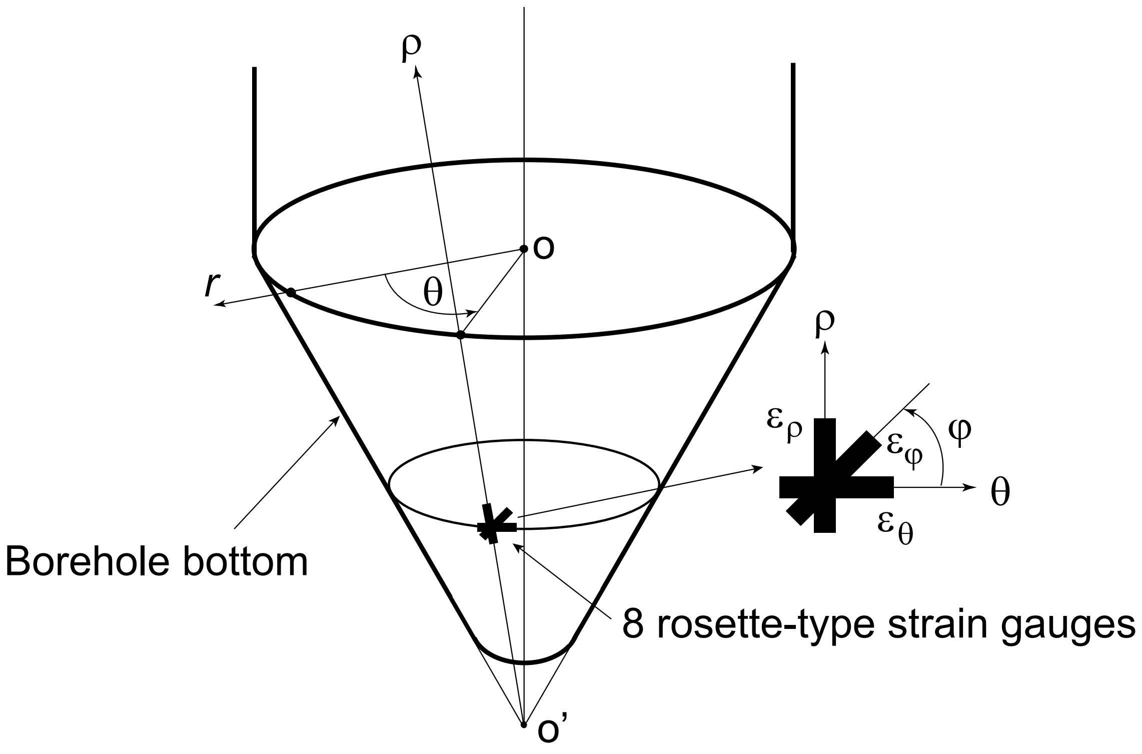

Supplementary Figure 2 Three typical directions of stains (ε_ρ, ε_θ, ε_φ) which are measured on the surface of the borehole bottom during overcoring .

(a)

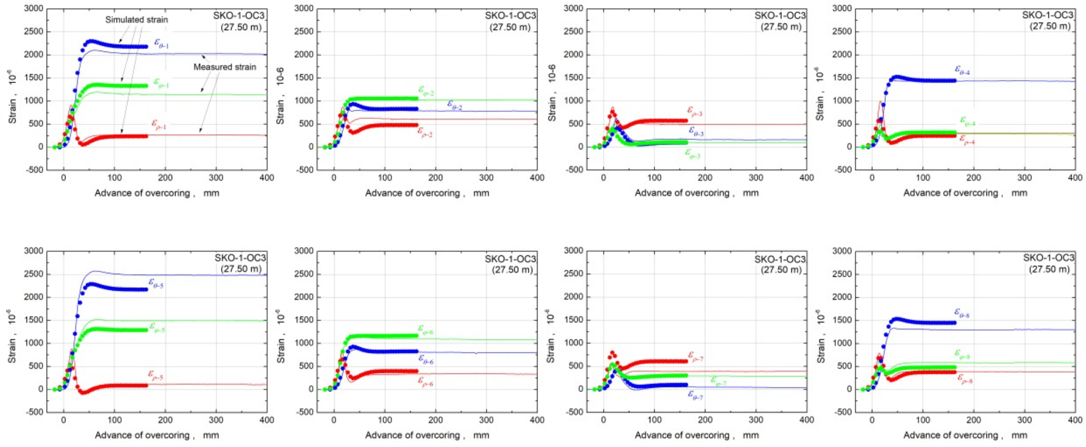
SKO-1-OC3
(27.50 m)
Simulated strain
Measured strain
Strain, 10⁻⁶
Advance of overcoring, mm

(b)

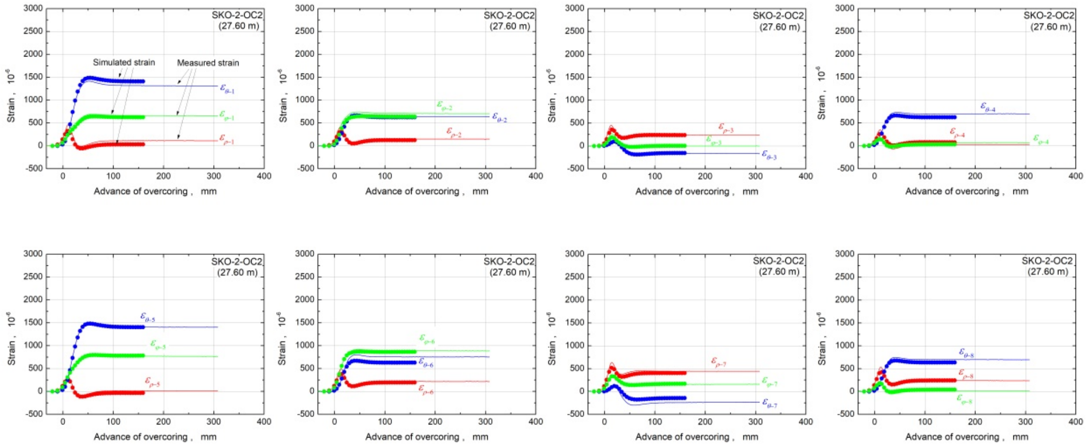
SKO-2-OC2
(27.60 m)
Simulated strain
Measured strain
Strain, 10⁻⁶
Advance of overcoring, mm

(c)

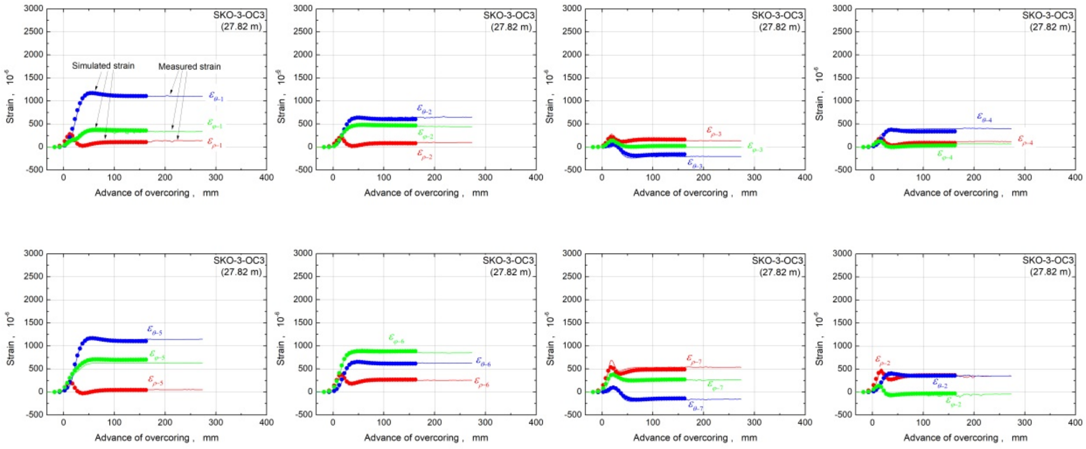
SKO-3-OC3
(27.82 m)
Simulated strain
Measured strain
Strain, 10⁻⁶
Advance of overcoring, mm

(d)

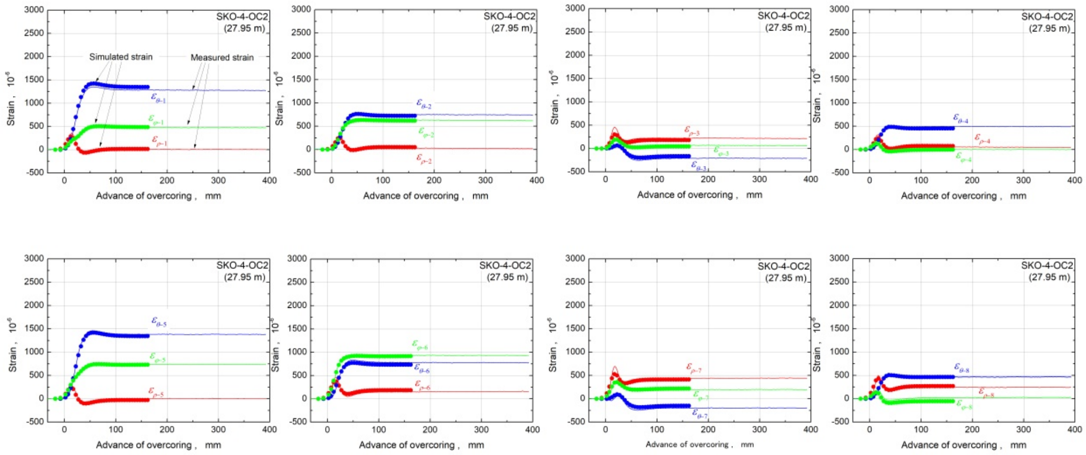

Supplementary Figure 3 The strain response during overcoring advance (solid lines), compared to the theoretical curves (solid circles). Theoretical curves were calculated based on the elastic theory using the evaluated stresses (see Supplementary Tables 3-6). BEM analysis was used to determine the components of the elastic compliance matrix for the different advance of overcoring [4]. (a) SKO-1-OC3, (b) SKO-2-OC2, (c) SKO-3-OC3, (d) SKO-4-OC2.

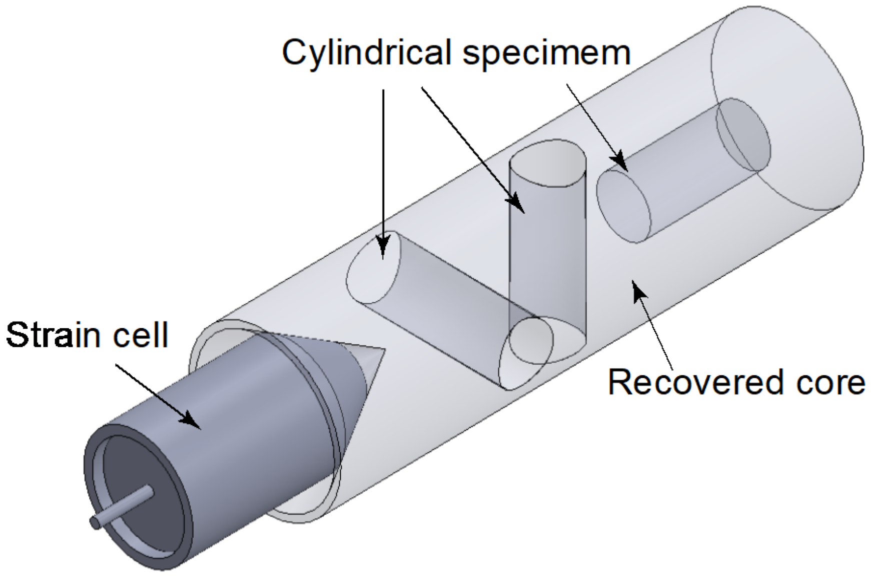

Supplementary Figure 4 Specimen sampling for the cyclic loading test.

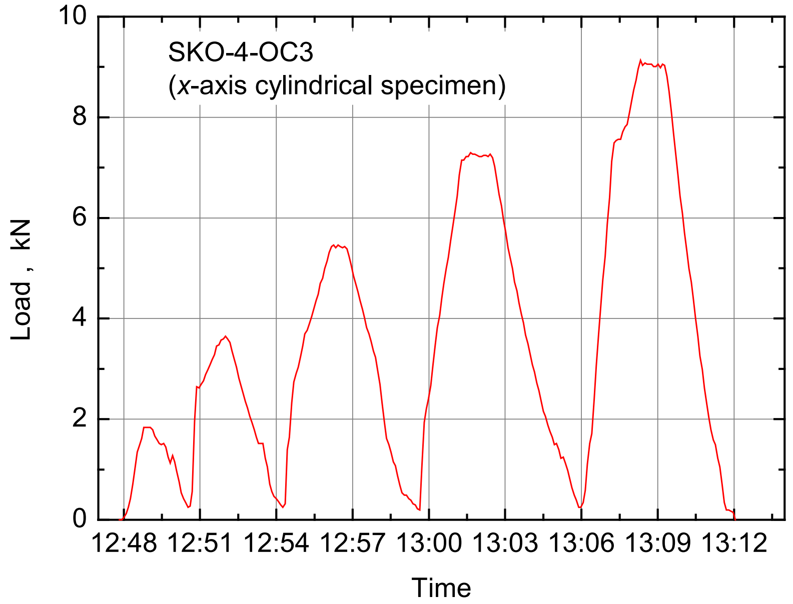

Supplementary Figure 5 An example of the loading pattern for the multi-stage uniaxial compression test. (*x*-axis cylindrical specimen of SKO-4-OC3)

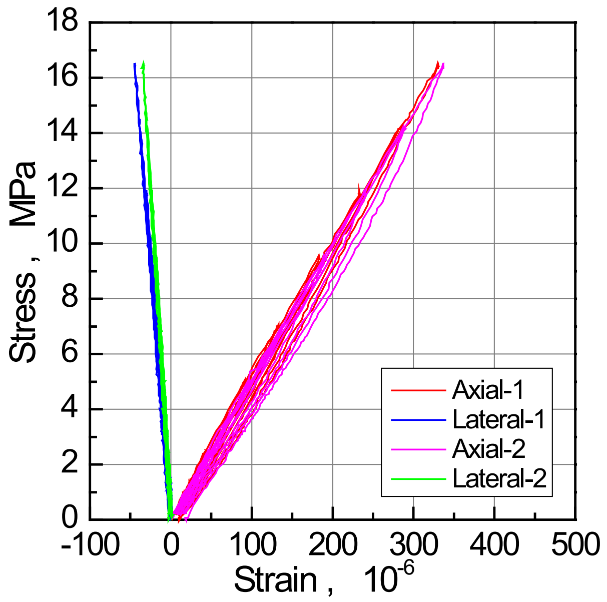

Supplementary Figure 6 An example of the stress–strain relation. (*z*-axis cylindrical specimen of SKO-1-OC1)

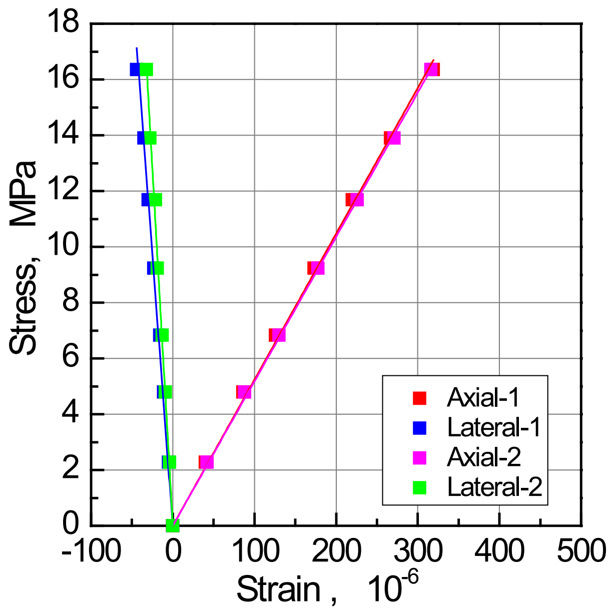

Supplementary Figure 7 An example of the relation between stress and strain recovery. (*z*-axis cylindrical specimen of SKO-1-OC1)

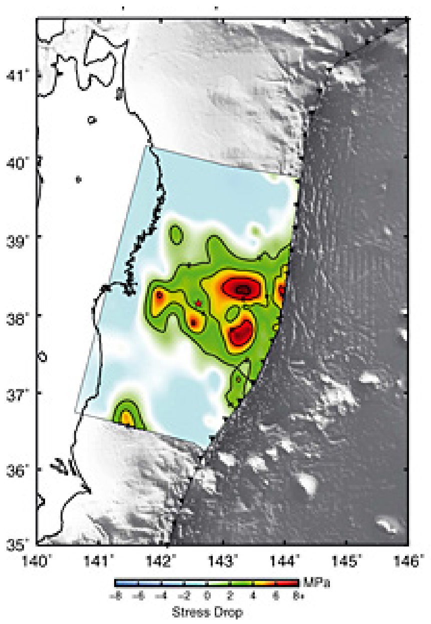

Supplementary Figure 8 Stress drop distribution due to the 2011 Tohoku-oki earthquake from Bletery et al [6]. The magnitude of stress drop at 53 km depth of the measurement station in the Kamaishi mine (39°19′N, 141°40′E) is negative 0.9MPa (by personal communication from Dr. Bletery).

Supplementary Table 1 Young's modulus and Poisson's ratio determined by multi-stage uniaxial compression tests. The values are the mean values of the Young's modulus and Poisson's ratio at each measurement point.

Recovered core	Young's modulus	Poisson's ratio
SKO-1-OC3	51.5	0.21
SKO-1-OC4	57.2	0.20
SKO-2-OC1	52.8	0.16
SKO-2-OC2	57.2	0.17
SKO-2-OC3	55.9	0.17
SKO-3-OC1	47.0	0.17
SKO-3-OC2	46.2	0.15
SKO-3-OC3	47.0	0.13
SKO-4-OC2	58.2	0.17
SKO-4-OC3	55.6	0.17
SKO-4-OC4	45.9	0.15

Supplementary Table 2. Magnitudes of the principal stresses (σ_1, σ_2, σ_3) and vertical stress (σ_v) before the earthquake and the overburden pressure (p_v).

	σ_1 [MPa]	σ_2 [MPa]	σ_3 [MPa]	σ_v [MPa]	p_v [MPa]	Reference
K-1	22.3	14.2	4.4	10.3	13.9	Ref. 2
K-2	29.3	7.6	2.9	4.7	7.0	Ref. 3
K-3	27.0	7.6	6.2	8.9	11.5	Ref. 4
K-4	25.0	8.0	6.6	7.9	6.4	
K-5	10.8	7.5	2.7	7.9	7.3	Ref. 5

Supplementary Table 3 Results of stress measurement in the SKO-1 borehole one year after the earthquake. (a) Results for the principal stresses (σ_1, σ_2, σ_3) and the overburden pressure (p_v). (b) Results for the stress component. E and N denote east and north, respectively.

(a)

SKO-1		σ_1	σ_2	σ_3	p_v
OC-3	Magnitude [MPa]	48.2	28.4	16.3	7.7
	Azimuth/Dip [°]	12.7/8.6	281.5/7.8	149.9/78.3	-
OC-4	Magnitude [MPa]	43.9	31.4	17.3	7.7
	Azimuth/Dip [°]	17.1/1.7	286.4/22.4	111.2/67.6	-
Mean±SD	Magnitude [MPa]	46.1 ± 2.2	29.9 ± 1.5	16.8 ± 0.5	7.7 ± 0.27
	Azimuth [°]	14.9 ± 2.2	283.9 ± 2.5	130.5 ± 19.3	-
	Dip [°]	5.2 ± 3.5	15.1 ± 7.3	72.9 ± 5.4	-

(b)

SKO-1	σ_E [MPa]	σ_N [MPa]	σ_v [MPa]	σ_{Nv} [MPa]	σ_{vE} [MPa]	σ_{EN} [MPa]
OC-3	29.2	46.5	17.2	−5.0	0.6	4.4
OC-4	30.7	42.5	19.4	−2.2	4.5	4.2
Mean±SD	30.0 ± 0.8	44.5 ± 2.0	18.3 ± 1.1	−3.6 ± 1.4	2.6 ± 1.9	4.3 ± 0.1

Supplementary Table 4 Results of stress measurement in the SKO-2 borehole two years after the earthquake. (a) Results for the principal stresses (σ_1, σ_2, σ_3) and the overburden pressure (p_v). The error of overburden pressure p_v was evaluated assuming that the measured value of overburden (depth) has an error of ±10 m. (b) Results for the stress component. E and N denote east and north, respectively.

(a)

SKO-2		σ_1	σ_2	σ_3	p_v
OC-1	Magnitude [MPa]	30.9	10.9	7.5	7.7
	Azimuth/Dip [°]	213.1/2.2	122.4/18.1	309.7/71.7	-
OC-2	Magnitude [MPa]	32.4	12.8	6.7	7.7
	Azimuth/Dip [°]	21.4/1.2	291.3/7.2	121.1/82.7	-
OC-3	Magnitude [MPa]	31.8	12.6	3.8	7.7
	Azimuth/Dip [°]	12.4/4.1	282.1/4.2	146.8/84.1	-
Mean±SD	Magnitude [MPa]	31.9 ± 0.4	12.0 ± 0.9	6.0 ± 1.6	7.7 ± 0.27
	Azimuth [°]	22.3 ± 8.5	291.9 ± 8.3	132.5 ± 10.7	-
	Dip [°]	2.5 ± 1.2	9.8 ± 6.0	79.5 ± 5.5	-

(b)

SKO-2	σ_E [MPa]	σ_N [MPa]	σ_v [MPa]	σ_{Nv} [MPa]	σ_{vE} [MPa]	σ_{EN} [MPa]
OC-1	16.7	25.1	7.8	1.3	−0.3	9.6
OC-2	15.3	29.8	6.8	−0.8	0.5	6.7
OC-3	13.5	30.8	4.0	−2.1	0.2	4.1
Mean±SD	15.2 ± 1.3	28.6 ± 2.5	6.2 ± 1.6	−0.5 ± 1.4	0.1 ± 0.3	6.8 ± 2.2

Supplementary Table 5 Results of stress measurement in the SKO-3 borehole three years after the earthquake. (a) Results for the principal stresses (σ_1, σ_2, σ_3) and the overburden pressure (p_v). The error of the overburden pressure p_v was evaluated assuming that the measured value of overburden (depth) has an error of ±10 m. (b) Results for the stress component. E and N denote east and north, respectively.

(a)

SKO-3		σ_1	σ_2	σ_3	p_v
OC-1	Magnitude [MPa]	19.7	9.2	3.0	7.7
	Azimuth/Dip [°]	216.2/1.4	126.0/7.4	317.1/82.5	-
OC-2	Magnitude [MPa]	22.6	9.7	3.8	7.7
	Azimuth/Dip [°]	219.3/4.3	129.2/2.1	13.2/85.2	-
OC-3	Magnitude [MPa]	21.6	12.1	4.1	7.7
	Azimuth/Dip [°]	197.4/2.4	107.2/5.1	312.7/84.3	-
Mean±SD	Magnitude [MPa]	21.3 ± 1.2	10.3 ± 1.3	3.6 ± 0.4	7.7 ± 0.27
	Azimuth [°]	211.0 ± 9.7	120.8 ± 9.7	325.5 ± 15.1	-
	Dip [°]	2.7 ± 1.2	4.9 ± 2.2	84.0 ± 1.2	-

(b)

SKO-3	σ_E [MPa]	σ_N [MPa]	σ_v [MPa]	σ_{Nv} [MPa]	σ_{vE} [MPa]	σ_{EN} [MPa]
OC-1	12.8	16.0	3.1	0.8	−0.4	5.1
OC-2	14.9	17.4	3.9	1.2	0.7	6.3
OC-3	12.9	20.7	4.2	0.9	−0.5	2.8
Mean±SD	13.5 ± 1.0	18.0 ± 2.0	3.7 ± 0.5	1.0 ± 0.2	−0.1 ± 0.5	4.7 ± 1.5

Supplementary Table 6 Results of stress measurement in the SKO-4 borehole five years after the earthquake. (a) Results for the principal stresses (σ_1, σ_2, σ_3) and the overburden pressure (p_v). The error of the overburden pressure p_v was evaluated assuming that the measured value of overburden (depth) has an error of ±10 m. (b) Results for the stress component. E and N denote east and north, respectively.

(a)

SKO-4		σ_1	σ_2	σ_3	p_v
OC-2	Magnitude [MPa]	31.7	9.8	5.8	7.7
	Azimuth/Dip [°]	205.2/4.3	295.2/0.6	33.7/85.6	-
OC-3	Magnitude [MPa]	33.0	13.1	5.0	7.7
	Azimuth/Dip [°]	191.2/3.0	281.8/10.7	85.9/78.9	-
OC-4	Magnitude [MPa]	24.0	10/.3	3.6	7.7
	Azimuth/Dip [°]	174.4/1.4	264.6/9.2	75.8/80.7	-
Mean±SD	Magnitude [MPa]	29.6 ± 4.0	11.0 ± 1.4	4.8 ± 0.9	7.7 ± 0.27
	Azimuth [°]	190.3 ±12.6	280.5 ± 12.5	72.9 ± 12.0	-
	Dip [°]	2.9 ± 1.2	6.8 ± 4.4	81.7 ± 2.8	-

(b)

SKO-4	σ_E [MPa]	σ_N [MPa]	σ_v [MPa]	σ_{Nv} [MPa]	σ_{vE} [MPa]	σ_{EN} [MPa]
OC-2	13.8	27.6	5.9	1.7	0.9	8.3
OC-3	13.5	32.2	5.4	1.1	1.7	3.8
OC-4	10.2	23.8	3.8	0.6	1.0	−1.4
Mean±SD	12.5 ± 1.6	27.9 ± 3.4	5.0 ± 0.9	1.1 ± 0.4	1.2 ± 0.4	3.6 ± 4.0

References

1. Japanese Geotechnical Society Method for initial stress measurement by compact conical-ended borehole overcoring technique, *JGS3551-1009* (2009).
2. Sakaguchi, K., Huang, X., Noguchi, Y. & Sugawara, K. Application of Conical-ended Borehole technique to discontinuous rock and consideration, *J. MMIJ* **111**, 283−288 (1995).
3. Japan Nuclear Cycle Development Institute, Final Report of Kamaishi In-situ Experiment, *JNC TN7410 99-001* (1999).
4. Sugawara, K. & Obara, Y. Draft ISRM suggested method for in situ stress measurement using the Compact Conical-ended Borehole Overcoring (CCBO) technique, *Int. J. Rock Mech. Min. Sci.* **36**, 307−322 (1999).
5. Sakaguchi, K., Takeda, H. & Matsuki, K. In-Situ rock stress measurement using improved Downward Compact Conical-Ended Borehole Overcoring technique, *J. MMIJ* **126**, 418−424 (2010).
6. Bletery, Q. *et al.* A detailed source model for the M_w 9.0 Tohoku-Oki earthquake reconciling geodesy, seismology, and tsunami records, *J. Geophys. Res.* **119**, 7636–7653, doi:10.1002/2014JB011261 (2014).
